# Why Computational Photochemistry Is Challenging and Will Probably Remain So: A Quantum Chemist's Perspective

**DOI:** 10.1002/advs.202521012

**Published:** 2026-03-12

**Authors:** Andreas Dreuw

**Affiliations:** ^1^ Interdisciplinary Center for Scientific Computing Heidelberg University Heidelberg Germany

**Keywords:** computational photochemistry, computational spectroscopy, electronic structure, excited states, quantum chemistry

## Abstract

Compared to ground‐state chemistry, the theoretical description of molecular photochemistry, that is, chemical processes in electronically excited states or electronic spectra, is fundamentally more complex. Even for relatively small molecules, several open decay channels have to be considered at the same time, and thus several electronically excited states with different electronic structures need to be computed. Furthermore, the required accuracy of the employed electronic structure theory increases with the size of the studied molecule, even for a qualitative investigation of photochemical processes. They critically depend on the correct relative energies of the electronic states and the details of the underlying potential energy surfaces (PES), and with increasing molecular size, the excitation energies become smaller and smaller, and more and more electronic states lie within the error margins of most common electronic structure methods. This imposes clear size limits for tractable molecular systems and introduces an early and fast transition from highly accurate calculations to wild guessing. Computational chemistry always lives with the lazy compromise between accuracy and computational effort, and this is particularly true for computational photochemistry, as will be discussed within this perspective from the point of view of electronic structure theory.

## Graphical abstract

Computational Photochemistry has made great strides in recent decades, but the investigation of larger molecules remains a challenge due to the inherent dilemma between the increasing computational accuracy required as the molecule size increases and the inevitable explosion in computational effort. Inevitably, Computational Photochemistry will thus still remain a challenge for the foreseeable future.

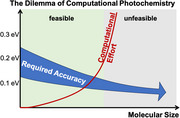

## 1 Introduction

Since the new Millennium Theoretical and Computational Chemistry has developed into an important pillar of chemical research of academia and industry alike. Nowadays, practically no larger chemical research endeavor is undertaken without consulting theory and computation to guide, for example, the synthesis of novel compounds or to support the interpretation of experimental spectroscopic data. Theoretical and Computational Chemistry have become indispensable in clarifying reaction mechanisms and predicting molecular properties, as well as for deriving new chemical concepts. Nowadays, they drive the understanding of chemistry.

In general, I like to differentiate between theoretical chemistry and computational chemistry in the sense that the former is concerned with the development and understanding of theoretical computational methods, while the latter is concerned with their application to chemical problems and their solution. Both are highly interdisciplinary and require knowledge of mathematics, computer science, physics, and, of course, chemistry in varying amounts.

The increase in importance of computational chemistry was certainly also due to the massive development and availability of computer technology that we witnessed over the last few decades. According to the so‐called Moore's Law [1], CPU speed increased exponentially and practically doubled every one to two years since the mid 1960s. However, this has come to an end [2, 3], and recent developments in computer technology focus on massive parallelization under the notion of high‐performance computing. The adaptation of computational chemistry methods to this development is an important research topic of its own [4, 5, 6, 7, 8, 9, 10, 11, 12], which, however, will not be covered here. In general, progress in Theoretical Chemistry, that is, the development of new, more efficient methods and algorithms, was at least as decisive, if not even more decisive, for the capabilities of Computational Chemistry than the technological development of computers. Indisputably, together they have made Computational Chemistry what it is today, an important, indispensable tool and field of chemical research in its own right.

Computational Chemistry is particularly successful in the field of thermal chemistry, in which reactions and processes take place on the potential energy surface of the electronic ground state. Usually, they thus proceed along well‐defined reaction pathways. For such problems, reliable predictions can be made for large molecular systems, for example, using density functional theory (DFT) [13]. For smaller systems, high accuracy can be achieved using coupled cluster‐based electronic structure methods such as CCSD[T] [14, 15], for example. In recent years, data‐driven machine learning (ML) has also been successfully applied in chemical research [16, 17, 18, 19]. In most cases, the training data for ML is generated by quantum chemical (QM) calculations with known accuracy for electronic ground states. However, accurate calculation methods are also required here for cross‐validation and quality assurance of the training data, as only high‐quality training data provides reliable ML‐based predictions.

The situation is generally different for Computational Photochemistry, which is the description of photo‐initiated chemical processes or the simulation of electronic spectra. In general, nuclear dynamics play a crucial role in fast photochemical processes, and thus, we will briefly touch upon them in the subsequent section. However, the reader is pointed to the recent review by Faraji et al. for an excellent overview and more detailed discussion of the state‐of‐the‐art of simulating nuclear dynamics in photochemistry [20].

In this perspective, the focus lies on electronic structure methods, since a correct and at the same time efficient computation of excited electronic state energies and their properties is a prerequisite for meaningful nuclear dynamics simulations. From an electronic structure point of view, photochemistry requires, in general, the simultaneous calculation of several excited states, at least the ground and first excited states, which is by itself already a daunting task. For the results to be useful, all relevant computed states should possess similar, in the best case, high accuracy to obtain reliable relative energies and spectra. The biggest dilemma of the theoretical description of photochemistry of larger systems is that the larger the molecules become, the higher the accuracy of the calculated excited states is needed, since energy differences become smaller and photochemistry depends on small details of the involved potential energy surfaces (PES). But only less accurate methods can be applied due to the required computational effort. We will discuss this quandary in more detail in Section 3.

When simulating photochemistry or electronic spectra, one aims at reproducing reality as closely as possible. In real experiments, photochemistry and spectra are usually observed in an environment, and gas‐phase experiments have become scarce, in particular for larger molecular systems. For a direct and fair comparison of computational and experimental results, one thus needs to consider the environment by virtue of appropriate models, which are obviously also sources of error. This aspect of modelling photochemistry will be discussed in Section 4. The main conclusions are summarized in Section 5. Lastly, this contribution is not intended as a comprehensive review, but as a critical discussion of and a personal view on computational photochemistry in general.

## 2 Electronic Structure Limits to Excited‐State Dynamics

**FIGURE 1:**
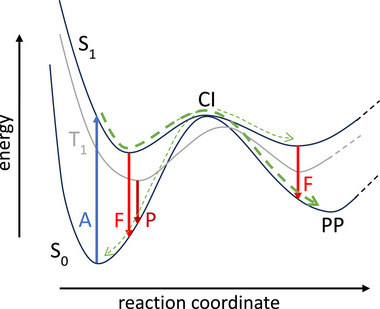
Sketch of the potential energy surface of a prototypical photochemical scenario, in which molecules are electronically excited by absorption (A) of light from the electronic ground state S_0_ to the first excited state S_1_. Decay occurs either radiatively via fluorescence (F) or phosphorescence (P) or non‐radiatively, often via a conical intersection (CI) to the photo‐product (PP) or other minima.

After absorption of visible, UV, or X‐ray photons, molecules are in an electronically excited state, that is, in a non‐equilibrium and thus decaying state, and the key task for understanding a photochemical reaction mechanism is thus to identify the main decay channels. This is not straightforward, because excited molecules generally possess several open decay channels: they can (i) fluoresce, (ii) decay non‐radiatively back to the ground state, (iii) undergo intersystem crossing into the triplet manifold and decay non‐radiatively, (iv) phosphoresce, or (v) perform some excited‐state reaction to a photoproduct in the singlet or triplet manifold (Figure 1). In reality, an ensemble of electronically excited molecules very often decays via several of these pathways simultaneously at different rates. Photochemistry is thus always a competition of timescales of various possible processes. Only if one pathway is significantly faster than all the others will this be the predominantly observed pathway and can be called “*the reaction mechanism*.” In general, to gain insights into the different rates of the decay channels, their lifetimes, and quantum yields, nuclear dynamics are necessary and thus generally crucial for a comprehensive and quantitative understanding of molecular photochemistry.

Practically all quantum and semi‐classical nuclear dynamics simulations rely on the separation of nuclear and electronic degrees of freedom, most prominently on the Born‐Oppenheimer approximation. Only then does the well‐known adiabatic picture of nuclei moving on potential energy surfaces (PES) emerge. In general, photochemistry involves nuclear motion, which proceeds on several, coupled PESs giving rise to non‐adiabatic chemistry. This is especially the case when conical intersections (CIs) are involved, where the Born–Oppenheimer approximation breaks down, and so‐called non‐adiabatic effects dominate. Today, it is well established that CIs are ubiquitous and usually determine the course of a photochemical reaction [21, 22]. They are thus rightly called “*photochemical funnels*.” Therefore, the correct location and topical description of CIs are key ingredients from electronic structure theory for nuclear dynamics simulations of photochemical processes, along with non‐adiabatic coupling elements, spin‐orbit‐coupling elements, and transition dipole moments, for instance [23]. Of course, errors in the electronic‐structure computations have an immediate impact also on the accuracy of the simulated nuclear dynamics, independent of the level of approximation of the latter.

When near‐exact solutions of the electronic structure problem can be computed, near‐exact nuclear quantum dynamics simulations are meaningful and worthwhile, since otherwise the errors in the PESs corrupt the accuracy of the costly simulation. When the studied molecules are, however, larger, only less accurate electronic‐structure data are available due to the limits of electronic structure theory (see below). Then, costly near‐exact quantum dynamics simulations are no longer sensible. Instead, computationally less demanding semi‐classical schemes like surface‐hopping dynamics are viable alternatives, of course, at the expense of less accuracy, since errors in electronic structure theory and dynamics may accumulate. Such nuclear dynamics simulations can, however, be very useful in exploring and discovering unexpected photochemical reactions, since the evolving dynamics can follow mechanisms unforeseen by the computational chemist.

Accordingly, the field of non‐adiabatic dynamics for the description of molecular photochemistry has made great progress over the last few decades owing to developments in electronic‐structure theory as well as nuclear dynamics simulation techniques. Trajectory surface hopping dynamics, particularly in combination with CASSCF, CASPT2, and related methods has become a cornerstone for the theoretical description of molecular photochemistry along CIs [24, 25, 26, 27, 28, 29]. Advanced trajectory‐based nuclear dynamics simulation techniques have been developed, incorporating quantum effects [30, 31, 32]. These developments allowed for the investigation of complex photochemical and photobiological processes [20, 23, 28, 29, 33, 34].

If one goes to very large molecules, for which only qualitative data are accessible by electronic structure theory, and maybe even the order of the excited states cannot be conclusively determined, performing any kind of quantum dynamics is practically useless. As a computational chemist, one is then usually better off to only visually inspect the PESs, since this can be sufficient to “*see*” how the photochemical reaction may proceed and to make tentative predictions about the photochemical processes. If this is the case, further support by either selected benchmark computations or experimental collaboration is needed to substantiate the proposed photochemical mechanisms.

It is therefore also the task of the resource‐conscious computational chemist to estimate when, what kind, and whether a simulation of the nuclear dynamics makes sense for the investigated photochemical process [20].

## 3 The Excited State Electronic Structure Problem

In contrast to thermal ground‐state chemistry, knowledge about several excited electronic states is needed for the description of photochemistry. It is clear that for the computed results to be useful, they should possess, in principle an as high an accuracy as possible, but at least similar accuracy for all calculated states to obtain reliable relative energies and spectra. As usual in computational chemistry, the choice of theoretical approach is a lazy compromise between accuracy and effort. Unfortunately, the required computational effort increases drastically with the size of the investigated molecules and quickly reaches hard limits of molecular size in terms of feasibility, in photochemistry, even much faster than in thermal ground‐state chemistry.

In ground state chemistry, the desired computational accuracy to make quantitative predictions is in general “chemical accuracy”, which is the typical experimental accuracy of 1 kcal/mol for relative energies and 0.001 Å for bond lengths, for example. Less accurate results lead, of course, to reduced predictivity, but are still useful for qualitative investigations. Remarkably, the required accuracy is independent of the molecular size in ground‐state chemistry. The situation is different for the investigation of photochemical processes, since the density of states in the low‐energy region of the electronic spectrum increases with molecular size and the excitation energies become smaller and smaller. Therefore, more and more computational accuracy is required for an unambiguous computational identification of the lowest excited state, or the correct description of CIs, the larger the investigated molecules become. In other words, the accuracy demand increases for computational photochemistry with increasing molecular size for quantitatively or even only qualitatively correct results.

In general, it is difficult to define clear accuracy limits for meaningful computational photochemistry, since they are always molecule‐specific and require careful individual consideration. Currently, a rule‐of‐thumb is that an accuracy of approximately 0.1–0.2 eV in excitation energies is adequate to reach near‐quantitative computational photochemistry in combination with appropriate nuclear dynamics simulations for predominantly organic molecules with up to 25 s‐ and possibly third‐row atoms of the period table, that is, 150–200 electrons. Such molecules typically exhibit excitation energies larger than 2 eV. This is certainly different for transition‐metal complexes with near‐degeneracies and also for larger molecules, in which the excitation energies become even smaller, and yet higher accuracy is needed.

The required accuracy also depends on the computational task. While the estimates above hold for electronic‐structure results as input for quantum dynamics simulations, that is, the PES as well as required transition properties, it can be very different for the simulation of UV–vis absorption spectra. In this case, only optically bright excited states are relevant, that is, a sub‐class of all excited states. In typical organic molecules, these are predominantly ππ^*^ excited states, in which valence electrons are excited from an occupied π‐orbital into an unoccupied π^*^‐orbital. Other states, like nπ^*^, πσ^*^, or long‐range charge‐transfer excited states, do usually not play a role, since they possess no significant oscillator strength and are thus not visible in the experimental spectrum. Therefore, for the modelling of absorption spectra, a QM method is needed that “just” yields the correct energetic spacing of the ππ^*^ states and their oscillator strengths. Even a substantial error larger than the said 0.1–0.2 eV can be unproblematic, if it is identical for all ππ^*^ states. In this case, one still obtains an accurately computed absorption spectrum with a constant energetic offset compared to the experimental one. The correct reproduction of a UV–vis spectrum very often serves as justification of an electronic structure method for its application in subsequent nuclear dynamics simulations. However, great care must be taken, since dark states possibly relevant for the nuclear dynamics are not benchmarked by reproducing an experimental absorption spectrum. In general, it is much more reliable to benchmark electronic structure methods for excited states among themselves, that is, by thorough comparison to the best possible, just yet affordable method.

Nowadays, there exists a plethora of different QM methods to calculate excited electronic states and their properties. In general, one distinguishes between single‐reference (SR) and multi‐reference (MR) methods. In SR methods, only one reference wavefunction is used to construct the excited state manifold, while in MR several references are used, which eventually allows to describe degenerate electronic ground states. This is particularly important for CIs between the ground S_0_ and the first S_1_ excited state. At such instances, SR methods do generally not converge and sometimes only collapse on an arbitrary solution at the intersection seam. It is thus clear that standard SR methods cannot be used to quantitatively describe radiationless decay back to the ground state along an S_1_/S_0_ CI.

The most prominent quantum chemical MR methods available today are complete active space self‐consistent field (CASSCF) [35, 36], its extension in second‐order perturbation theory (CASPT2) [37], as well as n‐electron valence state perturbation theory in second order (NEVPT2) [38], and variants thereof. These methods require the choice of an active orbital space within which excited‐state configurations are generated. When carefully and knowledgably applied, they are powerful methods, in particular in combination with the density‐matrix renormalization group (DMRG) algorithm [39], which allows for the choice of quite large active orbital spaces. Efforts are also undertaken to reduce the computational effort of CAS methods [36, 40, 41, 42, 43, 44]. However, the preselection of orbitals requires experience of the computational chemist and a priori knowledge about the relevant excitations. In general, it is very difficult to choose a balanced CAS space, in particular along excited‐state trajectories in dynamics simulations. For smaller molecules, CAS methods can reach near quantitative accuracy for excited states below 0.2 eV when the CAS space covers the relevant excitation space; however, for larger molecules with extended π‐systems, this is often not possible, and the errors increase quickly.

Multireference configuration interaction [45] is another important family of methods, which nowadays is typically used in combination with semi‐empirical molecular orbitals, as in AM1/MRCI or OM2/MRCI, building on the Austin Model 1 (AM1) [46] or the Orthogonalization Method 2 (OM2) [47] Hamiltonians. In recent years, the use of DFT orbitals in the DFT/MRCI [48, 49] scheme has gained attention due to its successes in the description of extended π‐systems and transition metal complexes [50, 51]. Multi‐reference coupled cluster (MRCC) [52] methods and multi‐reference algebraic diagrammatic construction (MRADC) [53, 54] schemes for excited electronic states have also been developed recently. However, they are only applicable to fairly small molecular systems, and computationally too demanding to be used in nuclear dynamics simulations.

The by far most widely used methods today for the computational investigation of photochemistry and electronic spectroscopies are SR methods, despite the failure of their standard variants in the description of CIs. They are primarily used to compute static optical absorption spectra at the ground‐state equilibrium structures, where CIs are usually not relevant, and to map out PES spatially distant from S_1_/S_0_ CIs. Sometimes reaction pathways are computed, and the PES is interpolated across such CIs to get a qualitative understanding of the possible reaction pathways.

Such wavefunction‐based methods comprise the equation‐of‐motion (EOM) and linear‐response (LR) coupled cluster (CC) schemes with singles and doubles (EOM‐CCSD and LR‐CCSD) [55, 56, 57, 58], approximate CC schemes of second and third order CC2 [59, 60] and CC3 [61] as well as the algebraic diagrammatic construction (ADC) schemes of second to fourth order ADC(2) to ADC(4) [62, 63, 64, 65, 66, 67]. All these methods are conceptually related, and the second‐order schemes possess typical errors of about 0.2–0.3 eV in excitation energies, while the third and fourth order schemes are slightly better with about 0.1 eV, however, at the expense of a drastically increased computational effort.

Linear‐response time‐dependent density functional theory (TDDFT) [68] computes excitation energies and transition properties as a response of the electronic density to an infinitely small perturbation by an oscillating electric field, and builds upon the Kohn–Sham density functional theory formalism [13]. Since its introduction [69, 70], TDDFT has become the most popular excited‐state method for medium‐ to large‐sized molecular systems with up to thousands of second‐row atoms [71]. The quality of the obtained results depends, however, as always in DFT‐based methods, on the choice of the exchange‐correlation (xc) functional, and different xc‐functionals work differently for different classes of excited states. The famous Jacob's ladder of xc‐functional approximations [72], leading rung by rung to the “*Heaven of Chemical Accuracy*” for the electronic ground state, is thus generally not transferable to excited states. For example, generalized gradient approximations (GGAs) work extremely well for excitation energies of local ππ^*^ excited states due to fortuitous error compensation, but yield physically wrong results for charge transfer (CT)‐excited states. The opposite is observed for long‐range separated xc‐functionals that improve the results for CT states drastically but often lead to increased errors in local excited states. In particular, the amount of non‐local orbital exchange contained in the xc‐functional has a large influence on the computed excitation energy and the properties of the excited states [73, 74]. Therefore, the right choice of xc‐functional depends on the chemical question to be answered computationally, and requires, in any case, careful benchmarking against higher levels of theory, as those mentioned in the previous paragraphs.

**FIGURE 2:**
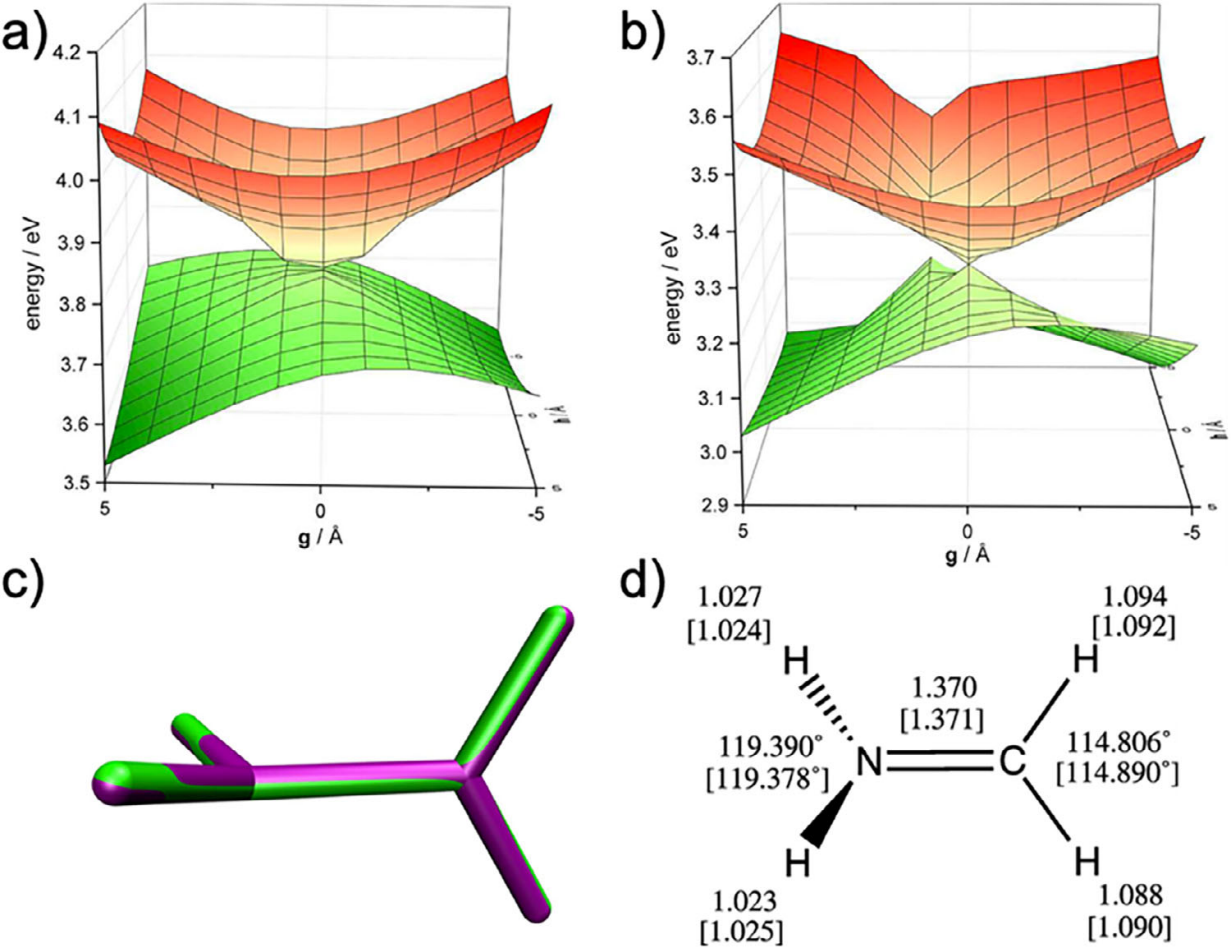
Comparison of the 2D plots of the S_1_ and S_0_ PESs in the vicinity of the conical intersection along the twisting coordinate of the formaldinium cation at the level of (a) SF‐ADC(3) and (b) MS‐CASPT2. An overlay of the optimized structures at SF‐ADC(3) level (green) and MS‐CASPT2 (purple) is shown in (c), and a comparison of the geometrical parameters (MS‐CASPT2 values in brackets) is made in (d).

All these SR methods yield reliable absorption spectra for SR ground state molecules around their equilibrium geometries, but they are not suitable for excited state dynamics, since their calculated PESs exhibit prohibitively large errors as soon as MR character is relevant for the electronic ground state. This is typically the case when geometries close to ground state CIs are reached, or bonds dissociate, which are very common situations in photochemical reactions. A viable approach to make SR methods also work in these situations is the so‐called spin‐flip formalism [75, 76]. The key trick is to choose the high‐spin triplet state (S = 1, m_S_ = 1) as a single reference instead of the singlet ground state (S = 0, m_S_ = 0). All excited‐state determinants are then created by replacing occupied orbitals with virtual ones with one flipped spin. Application of this procedure generates all excited singlet states (S = 0, m_s_ = 0), including the singlet ground state, as well as all triplet states (S = 1, m_s_ = 0), which are then treated on the same footing. This clever spin‐flip approach enables the investigation of singlet and triplet diradicals, singlet‐triplet gaps, the topography of CIs (Figure 2), and excited‐state reaction pathways using SR methods. At present, spin‐flip implementations of various excited‐state methods exist: SF‐EOM‐CC [75], SF‐ADC [77], SF‐TDDFT [78]. These simple tweaked SF methods possess, however, the drawback that they often suffer from severe spin contamination, which can be cured by spin‐adaptation procedures [76]. It is important to recognize that SF approaches require a stable SR triplet wavefunction, which limits their applicability to cases in which only two electrons are decoupled, which is typically the case when one single bond is broken or a double bond is rotated.

For the simulation of typical X‐ray spectroscopies [79], only singly core‐excited states are relevant and need to be computed, in which one core electron is excited into a valence orbital. These states have essentially SR character, and their excitation energies are two to three orders of magnitude larger than those of typical valence‐excited states. They are energetically well separated and practically do not couple to either doubly core‐excited states or valence‐excited states. This is exploited within the core‐valence separation (CVS) approximation [80], which allows for the development of computational methods particularly designed to compute singly core‐excited states. It has been successfully employed for EOM‐CC [81, 82], ADC [83], CASSCF [84], and TDDFT [85, 86], for example. While methods like the frozen core (FC) CVS‐EOM‐CCSD and extended CVS‐ADC(2)‐x reach accuracies of benchmark methods, CVS‐TDDFT often yields large errors in absolute excitation energies but still accurate energetic shifts and thus correct spectral shapes for typical organic molecules [87].

Briefly summarizing this paragraph, many excited‐state electronic structure methods exist, multi‐ and SR methods, based on wavefunction or density functional theory, possibly tailored for specific purposes. However, none of them is a one‐fits‐all method, since all methods applicable to larger molecules require approximations, making the calculations feasible. These approximations inevitably influence different classes of states differently due to their varying electronic structures and thus introduce imbalances into the electronic excitation spectrum. Therefore, it is recommended to always use all applicable and available excited‐state methods, different flavors of xc‐functionals within TDDFT, and to extrapolate from the comprehensive results, knowing the individual strengths and weaknesses of the methods onto the chemical reality.

## 4 Approximating Real Photochemistry, Real Spectroscopy

The goal of computational photochemistry is to understand photochemical processes, help interpret experimental spectra, and to eventually predict photochemical properties of new molecules. Experimental investigations and real‐life photochemical processes typically occur in some environment of the initially excited molecule. This can be, in the easiest case, the gas phase, but for larger molecules, more likely a solution, a film, or a protein. While in silico calculations of isolated molecules are directly comparable to gas‐phase experimental data, all other environments have a direct influence on the excited electronic states and the resulting photochemistry and spectra. This needs to be considered when computational results are to be compared with experimental data.

Since molecular excitations are usually local events occurring mostly on one excited molecule, or as in charge‐transfer or in excitonically coupled excitations on typically two, a molecular perspective is very often justified in the computational setup. Then the full molecular system can be separated into a photoactive molecular site and the environment, which allows for treating the different parts at different theoretical levels with an appropriate coupling between them. It is important, though, to include all molecules participating in the excitation and photochemical process within the photoactive site and to treat them together in the QM excited‐state calculation. The environment can then either be treated at a lower level of theory or be modeled implicitly or explicitly according to the specific situation.

For molecules in solution, polarizable continuum models (PCM) have been developed [88, 89, 90], assuming the atomistic nature of the solvent can be neglected, and the linear response is sufficient to describe the dielectric polarization. Then the solvent can be treated as a polarizable continuum employing the macroscopic dielectric constant as the only crucial input. They work the better the interaction between the solute and the solvent molecules are. If strong interactions are present, it is advisable to include solvent molecules explicitly in the QM calculation. In the context of computational photochemistry, different flavors of PCMs exist depending on the computational task. When absorption spectra are to be computed, a linear response approach to compute the influence of the solvent on all excited states is advisable [91, 92]. When, however, photochemical reactions or fluorescence spectra are to be computed, the solvent can adapt to the electronic structure of the excited state, and then state‐specific formulations of PCMs are required [93, 94].

As soon as the atomistic structure of the environment plays a role in the photochemistry, as is typically the case for photoactive molecules in proteins or in solids, that is, in static or slowly moving environments, PCMs are usually not appropriate and lead to substantial errors. Then, a structurally resolved model for the environment is mandatory. These methods are usually summarized under the notion of quantum mechanics/molecular mechanics (QM/MM) methods. Until today, different variants of such QM/MM coupling schemes have been devised, which differ in their treatment of the interaction between QM and MM subsystems. They are distinguished as electrostatic and polarizable embedding schemes [95, 96, 97]. In the simplest electrostatic QM/MM embedding, just point charges approximate the atoms of the environment, and the classic electrostatic interaction is included. More involved are frozen‐density embedding schemes [98, 99, 100], in which the interaction with a separately computed frozen density, typically at the DFT level, is taken into account in the excited state calculation [101]. Here, more than just the classical electrostatic interaction is considered, but the density of the environment is still not polarizable. For that, a freeze‐and‐thaw strategy is often employed [98].

Other polarizable QM/MM schemes comprise the effective fragment potential approach or polarizable embedding, which have also been realized recently, and efficient computer programs have been provided [102, 103, 104, 105, 106, 107, 108].

From the perspective of the computational photochemist, the choice of a proper environment model is as important for quantitative and even reliable qualitative results as the correct choice of the electronic structure theory, since the inherent errors can be of similar magnitude, and one should and can, in general, not rely on their fortuitous compensation. In principle, the same conclusions hold for the environment model as for the level of electronic structure theory. The larger the molecules become, the more accurate the environment model should be, since small errors have a larger influence on small excitation energies of large molecules than on large excitation energies of small molecules. At the same time, the accuracy of the electronic structure model and the employed environment model should be balanced, as it is rather pointless to ruin the accuracy of one by using an inaccurate other.

## 5 Quo Vadis Computational Photochemistry?

Computational photochemistry has made great progress over the last few decades due to enormous advances in computer technology, but much more so due to the efforts in the development of excited‐state electronic structure methods and approximate nuclear dynamics simulation techniques. However, its progress still seems to lag behind the advances in computational ground state chemistry, when the tractable molecular sizes and the accuracies achieved are considered. This apparent lag is due to the peculiarities of photochemistry itself, which requires an increasing accuracy of excitation energies and PES with increasing molecular size in order to provide a quantitatively and sometimes even qualitatively correct theoretical description. That's why there is a sharp line between highly accurate results, brilliant computational photochemistry, and total nonsense and useless results. To not cross this sharp line, expert knowledge in theoretical methods and photochemistry is most certainly warranted, as well as thorough benchmarking and/or experimental backup are always needed. In general, predictive computational photochemistry is very difficult and only possible for smaller molecules possible

From an electronic‐structure methods point of view, until today, no one‐fits‐all‐purpose excited‐state method exists that is applicable to all sorts of molecules with more than a few second‐row atoms. It is thus strongly advisable to use all available and applicable approximate methods: multi‐ and SR methods, wavefunction‐ or density‐based approaches, semi‐empirical methods, and to analyze the results carefully. Knowledge of the inherent and unavoidable errors of the different methods allows the expert computational photochemist to extrapolate to reality and to help interpret experimental spectra and to study photochemical mechanisms. Thereby, an important contribution to the understanding of photochemistry can be made despite the inherent limits of computational photochemistry.

Nowadays, a trend toward the adaptation of excited‐state electronic structure methods to high‐performance computing environments is observed, which allows for the exploitation of the ever‐growing computational resources. However, this requires especially tailored software utilizing highly parallel algorithms. In general, accurate wavefunction‐based excited state methods are substantially more difficult to parallelize than less accurate density‐based methods, and the latter suffer from the same dilemmas for larger molecules, also in HPC environments. Recently, ML methods have been used to learn PES from training data provided by electronic structure calculations, and thereby to accelerate nuclear dynamics simulations. In fact, this enables the simulation of longer timescales; however, the quality of the learned PES depends strictly on the accuracy of the training data. Since the training data is generated using QM methods as described above, ML exhibits the same accuracy limitations, and the question always remains whether its accuracy suffices for meaningful simulations of molecular photochemistry.

Also, the advent of the first quantum computers promises a substantial speed‐up of QM calculations in general via the quantum effect [109, 110]. Accordingly, several algorithmic approaches also for photochemical applications, that is, electronic structure calculations in combination with non‐adiabatic dynamics, have already been devised [111, 112, 113]. However, quantum computer technology is still in its infancies and whether it arrives at a level of everyday, broadly accessible computing infrastructures similar to the existing technology for solving photochemical questions in daily chemical research is still questionable.

Therefore, I cannot foresee an immediate methodological development that cures all the problems and that will provide a one‐fits‐all solution. In the near future, computational photochemistry will thus remain a challenge for theoretical chemists, and never a black box, as it requires expert knowledge of the applied methods and, at the same time, expertise in photochemistry. Fortunately, not every photochemical problem requires a highly accurate answer, and thus computational photochemistry is capable of investigating real photochemical problems, despite its still existing limitations and inherent dilemmas between molecular size and required accuracy.

## Conflicts of Interest

The authors declare no conflict of interest.
